# Characterizing Flow and Structure of Diabetic Retinal Neovascularization after Intravitreal Anti-VEGF Using Optical Coherence Tomography Angiography: A Pilot Study

**DOI:** 10.1155/2021/2942197

**Published:** 2021-07-14

**Authors:** Christof Haensli, Katrin Fasler, Daniel Barthelmes, Sandrine A. Zweifel

**Affiliations:** ^1^Department of Ophthalmology, University Hospital and University of Zurich, Zurich, Switzerland; ^2^Save Sight Institute, University of Sydney, Sydney, New South Wales, Australia

## Abstract

*Background*/*Aims*. This study evaluates changes of flow and structure of diabetic retinal neovascularization (NV) treated with intravitreal antivascular endothelial growth factor (VEGF) agents using optical coherence tomography angiography (OCTA). With OCTA, retinal blood vessels are visualized at high resolution to separately look at flow and structure information without the need for dye injection. We introduce a new measurement method including and combining information of flow and structure. *Methods*. Retrospective observational case series. Patients with proliferative diabetic retinopathy (PDR) were treated with intravitreal antiVEGF injections. Retinal NV were repeatedly imaged using swept-source OCTA (Zeiss PlexElite 9000) at baseline, after initial treatment block with 3-4 monthly injections, and during a follow-up period of up to 51 weeks. Change of size and flow density of the structural and angio area of NV was assessed. *Results*. Nine NV in eight eyes of five patients were analyzed with a median follow-up time of 45 weeks. After the initial treatment block, en face structural area regressed, 18.7% ± 39.0% (95% CI 44.2–6.8%, *p*=0.26), and en face angio area regressed, 51.9% ± 29.5% (95% CI 32.6 to 71.2%, *p*=0.007). Flow density within the en face structural area decreased by 33% ± 19.2% (95% CI 20.5–45.5%, *p*=0.0077). Flow density within the en face angio area decreased by mean 17.9% ± 25.2% (95% CI 1.4–34.4%, *p*=0.066). In two fellow eyes, NV recurrence could be observed before the onset of vitreous bleeding in one. *Conclusion*. Our study introduces a new quantitative measurement for NV in PDR, combining structure and flow measurement. The structure area remained after treatment, while its flow density and angio area regressed. We propose this measurement method as a more physiological and possibly more comparable metrics.

## 1. Introduction

Diabetic retinopathy (DR) is a leading cause of vision loss and blindness worldwide and presumably on the rise with expected demographics [1, 2]. Specifically, proliferative diabetic retinopathy (PDR), characterized by retinal neovascularization (NV), is responsible for severe visual impairment (e.g., due to vitreous hemorrhage or tractional retinal detachment) [3]. The mainstay of treatment has been panretinal photocoagulation (PRP) for almost 50 years [4]. However, PRP is associated with significant side effects, such as (contrast) vision loss, restriction of visual field, and development and worsening of macular edema [5, 6].

Antivascular endothelial growth factor (VEGF) is currently emerging as a promising treatment alternative for PDR [7–10]. Two multicenter, prospective clinical trials showed noninferior visual acuity (VA) results of intravitreal anti-VEGF (CLARITY for aflibercept; Protocol S for ranibizumab) compared to PRP [7, 9]. The CLARITY trial even showed improved VA, better treatment satisfaction scores, lower incidence of center-involving macular edema and vitreous hemorrhage, and less visual field loss with aflibercept than PRP [7]. Five-year data of Protocol S recently reported sustained noninferior VA outcomes and lower incidence of macular edema in the ranibizumab group [8]. Both of these studies have made treatment decisions about anti-VEGF injection based on clinical and photographic assessment of activity of NV [7, 11].

Optical coherence tomography (OCT) has been shown to be superior to the clinical detection of neovascular changes in PDR [12]. Recently, OCT Angiography (OCTA) has been used to further characterize NV with the aid of flow information additive to the structural OCT image [13–17]. Few studies have analyzed changes of NV in PDR after treatment with OCTA to date, and mainly on a short-term basis [17–24]. Significant advantages of OCTA are the practical repeatability due to its speed and noninvasiveness, the combination of flow information (similar to conventional fluorescein angiography (FA)) and structural information (OCT image), and the possibility of quantitative analysis. The aim of this pilot study is to introduce a new method of quantitative analysis of diabetic retinal neovascularizations. We use OCTA to measure, link, and assess structural and flow changes of NV in eyes with PDR undergoing treatment with anti-VEGF.

## 2. Methods

### 2.1. Ethics

Institutional review board approval (Ethics Committee of the University of Zurich, BASEC-No. PB_2016-00264) was obtained and all patients gave informed consent to publish their clinical data. The study adhered to the tenets of the Declarations of Helsinki.

### 2.2. Study Population

This study is a single-center, retrospective observational case series of patients diagnosed with PDR, confirmed on clinical exam and FA with or without previous treatment for DR (i.e., PRP, anti-VEGF, and vitrectomy >3 months from baseline) who were treated with anti-VEGF. Measurements were made on the basis of clinical needs. Data were included from April 2018 until September 2019 from patient records. Exclusion criteria were other causes of proliferative retinopathy (e.g., retinal venous occlusions and ocular ischemia), neovascular glaucoma, media opacities precluding good quality imaging of less than 7/10 in the manufacturers quality index (e.g., advanced cataracts and dense vitreous hemorrhage) or NV not accessible for quantitative measurements, and patients with combined treatment consisting of PRP and anti-VEGF. Patients with a follow-up of less than 4 months were excluded from the study.

All patients underwent a comprehensive ophthalmic examination at baseline including best-corrected VA measured in Early Treatment Diabetic Retinopathy Study (ETDRS) letters or Snellen decimal, intraocular pressure, slit lamp examination, dilated fundus examination using indirect ophthalmoscopy, widefield FA, spectral-domain OCT (Heidelberg Spectralis, Heidelberg Engineering, Heidelberg, Germany) of the macula, and swept-source OCTA (Zeiss PLEX® Elite 9000, Zeiss Meditec, Dublin, California, USA) scans of all detected neovascularizations for which OCTA achieved sufficient image quality for quantitative analysis. Scanning patterns of 3 × 3 mm, 6 × 6 mm, 12 × 12 mm, or 9 × 15 mm were chosen depending on size, location, and visibility.

### 2.3. Treatment and Follow-Up

Patients diagnosed with active PDR were informed about treatment options, including PRP or intravitreal anti-VEGF (aflibercept or ranibizumab), and involved in the treatment decision based on their respective needs and preferences. Patients with small NV on FA, which were hard to detect using indirect ophthalmoscopy, received repetitive OCTA imaging, as needed for clinical decision-making. Treatment consisted of an initial treatment block of three to four monthly anti-VEGF injections with ranibizumab or aflibercept. Further follow-up was carried out on the basis of the clinical course of the disease, with intervals of four to ten weeks. Further treatment was indicated as needed in the case of recurrent active proliferative diabetic retinopathy or visual impairment due to diabetic macular edema. The retreatment decision was based on indirect ophthalmoscopy and OCTA imaging as needed.

All NV were recorded at least at baseline and one month after the initial treatment block (posttreatment). For analysis, also the last available follow-up OCTA measurement was included (last follow-up). The in-between measurements were included in the longitudinal graphs.

### 2.4. Image Processing

**Figure 1:**
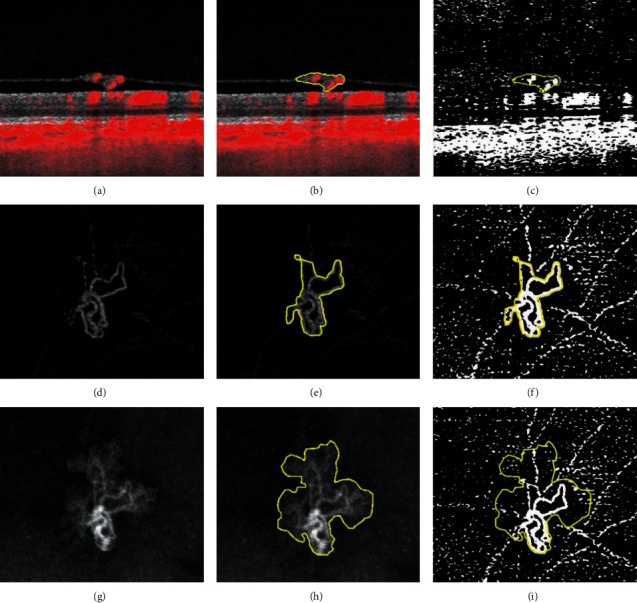
Summary of image processing steps. The first column shows the images generated by the optical coherence tomography angiography; a B-scan structure image with flow overlay in red (a), an en face flow (angio) image (d), and an en face structural (structure) image (g). Borders of the neovascularization were manually delineated for each image (second column: (b, e, h)), and the areas were measured. After binarization of the flow information, flow density was measured in percentages of white pixels within the respective area (last column: (c, f, i)).

Image analysis and quantitative evaluation were performed separately for en face and B-scan images. The manufacturer's OCTA software (Zeiss PLEX® Elite Review-Software, Zeiss Meditec Inc., Dublin CA, USA) provides B-Scan OCTA images consisting of a black and white structural image with flow overlay in red (Figure 1(a)). En face images of the vitreoretinal layer were generated for the flow (angio) and the structural (structure) image (Figure 1(d) for angio and Figure 1(g) for structure). The vitreoretinal interface layer (VRI) starts at the internal limiting membrane (ILM) and includes the vitreous cavity as far as the NV reached. The automated retinal layer segmentation provided by the software was manually checked and adjusted where necessary to smoothly follow the plane of the ILM underneath the protruding NV. En face images of the angio and structure images of the VRI layer were built using maximum intensity projection and exported as tagged image file format (TIFF) for further analysis. B-scan image stack with flow overlay was manually searched for the section with the highest projection of the NV towards the vitreous space. The height of maximum projection from the ILM towards the vitreous cavity of the NV was manually measured for both structure and flow information within the manufacturer's software. The respective image with flow overlay was exported as a TIFF file for further analysis.

### 2.5. Image Analysis

Image analysis was performed using the open source Fiji software (https://imagej.net/Fiji version 2.1.0/1.52) [25]. In the en face images, NV were manually delineated in the angio and the structure image separately, resulting in separate dimensions of the NV in angio (NV-angio) and structure images (NV-structure) (Figure 1(e) for angio and Figure 1(h) for structure). As necessary, the referenced B-scan image stack was used to guide demarcation in the usually low contrast structure slab. The area of the NV in the angio and structure images was measured. The angio images were binarized using the Phansalkar auto local threshold method (Figures 1(c), 1(f), and 1(i)) [26]. Flow density (FD) was defined as the percentage of white pixels after binarization. Flow density was measured in the binarized angio images within borders of NV-angio (FD-angio) and NV-structure (FD-structure) separately (Figure 1(f) for FD-angio and Figure 1(i) for FD-structure). Sequential measurements were performed during treatment and every available follow-up over time.

In the selected B-scan, the structural borders of the NV were manually delineated, the borders defined by the ILM and the vitreous space (Figure 1(b)). Subtraction of blue from red color information was performed after color splitting of the image, revealing the isolated flow information (red in the exported RGB-image file). The result was binarized using the Phansalkar auto local threshold method [26]. This method resulted in isolated binarized flow information in the respective B-scan with flow information in white (Figure 1(c)). Flow density was defined as the percentage of white pixels within the NV area. Area of the NV and FD within the NV area was measured.

### 2.6. Outcome Measures

Primary outcomes were changes of FD in the en face structure and angio area and changes of the size of NV-angio area and NV-structure area in the en face images at baseline, 5 ± 1 weeks after the initial treatment block with three to four monthly injections and at the last follow-up. Secondary outcomes were changes of height, area, and FD in the B-scan images. Visual acuity measurements and complications during observation time were recorded.

### 2.7. Statistical Analysis

Data of en face images for NV sizes in angio and structure imaging (NV-angio and NV-structure) and flow density within both areas (FD-angio and FD-structure) are presented as median with range and presented as box-plot graphs for changes from baseline to posttreatment (one month after initial treatment block) and last follow-up. Longitudinal graphs allow for some comparability of the in-between measurements, with weeks in the *x*-axes and scales for the *y*-axes standardized to set baseline size as 1 for areas and percentages for flow density. Data from B-scan imaging are summarized as longitudinal graphs in the supplementary material. Data were analyzed and visualized using Python Version 3.6 with Pandas library Version 1.2 and Microsoft® Excel for Mac, Version 16.47.1. Wilcoxon signed-rank test was used for calculation of p-values of measurements, compared to baseline. Confidence intervals were calculated for mean differences from baseline as 95% CI.

## 3. Results

### 3.1. Study Cohort

Eleven NV in nine eyes of six patients were treated as described. One patient was lost to follow-up. Nine NV in eight eyes of five patients were included in this study. Five patients were treatment-naïve PDR with type 2 diabetes; one eye had high-risk diabetic retinopathy with minor vitreous hemorrhage. In three patients, both eyes with one NV each were included. In one patient and two NV of one eye were included. One patient (two eyes with one NV each) had type 1 diabetes and had previous PRP treatment in both eyes, three months before baseline in one eye and ten months before baseline in the other eye. At baseline, no DME was present. The initial treatment block included three injections for eight eyes and four injections for three eyes of two patients. The median of the last OCTA follow-up period was 45 (range 19–51) weeks. Both eyes of one patient received three additional monthly anti-VEGF injections 37 weeks from baseline due to recurrent PDR, as described in the case presentation. One eye of the patient with type 1 diabetes received two anti-VEGF injections 27 and 45 weeks from baseline due to diabetic macular edema, while on OCTA, no NV was detectable.

### 3.2. NV Changes

#### 3.2.1. En Face NV Area in Structure and Angio Slabs

**Figure 2:**
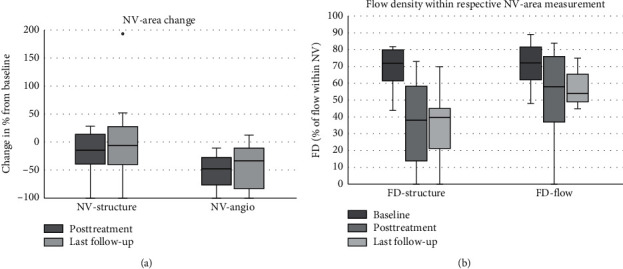
(a) Changes of neovascularization- (NV-) size measurements relative to baseline after the initial treatment block of three to four intravitreal anti-VEGF injections, and at the last follow-up. En face structural area (NV-structure) decreased by median 15% (range −28%–100%) and remained decreased by median 6% (−196%–100%). En face flow area (NV-angio) decreased by median 48% (range 10–100%) and remained by median 34% less than baseline (−13%–100%). The graph shows the reduction of NV size is more prominent in the angio than the structural en face OCTA image. (b) En face flow density (FD) measurements within the structural neovascularization (NV-area (FD-structure) and the flow NV-area (FD-angio)) at baseline, after the initial treatment block of three to four intravitreal anti-VEGF injections, and at the last follow-up: FD-structure started at median 64% (range 41–82%), decreased to median 32% (0–73%), and remained at median 32% (0–70%). FD-angio started at median 71% (range 48–89%), decreased to median 51% (0–84%), and remained at median 54% (0–75%). The graph shows that the difference in flow density between baseline, after treatment, and with apparently quiescent diabetic retinal neovascularization is more pronounced within the structural than the angio area of a NV.

**Figure 3:**
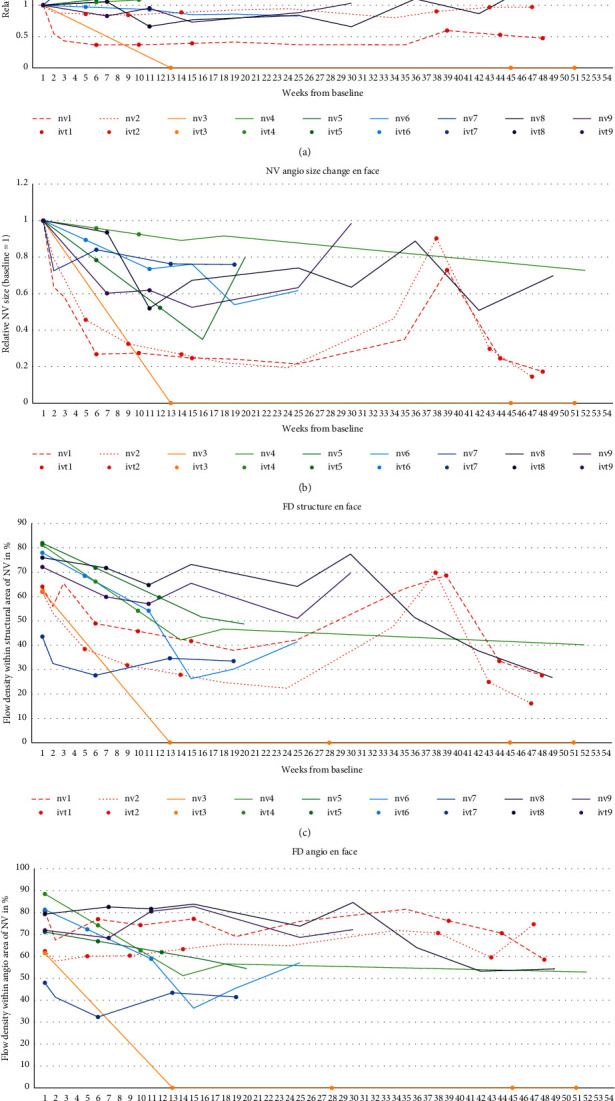
(a–d) Visualizing changes of area size and flow density of neovascularization (NV) for the flow and the angio area for every NV separately (data series NV 1–9). Bullet points represent time points of antivascular endothelial growth factor (anti-VEGF) injections in the respective eyes (data series IVT 1–9). It is indicated that in en face imaging, the structural framework (NV-structure) is at least partly remaining despite the fact that perfusion area (NV-angio) and flow density within the structural area (FD-structure) are regressing, while flow density within the angio area (FD-angio) is relatively stable. The two NV from the case presentation (NV 1 with vitreous hemorrhage and NV 2) are represented in dotted lines for better recognition. (a) Relative change of NV-structure from baseline (=1). The graph shows that the structural areas of the NV decrease only slightly. (b) Relative change of NV-angio from baseline (=1). The graph shows that in most NV, the angio area en face is decreasing after treatment with anti-VEGF. Two NV are growing back to a significant proportion after a long treatment-free interval. In one of these eyes, a new vitreous hemorrhage occurred before retreatment. (c) Flow density within the structural NV-area (FD-structure) in %. The graph indicates the consistent decrease of flow density within the structural areas of the NVs after treatment, as well as a new increase of the two NVs with recurrent proliferative diabetic retinopathy (PDR) activity. (d) Flow density within the angio NV-area (FD-angio) in %. The graph indicates the lesser decrease of flow density within the structural areas of the NVs after treatment. Also, in the case of the two NVs with recurrent PDR activity, it cannot be determined by the flow density of the NV measured on the OCTA angio image.

The neovascularization en face area showed different changes for structure and flow area (see Table 1 and Figure 2(a)). As visible in the longitudinal graph, no general trend of en face NV-structure regression can be observed over all NV (Figure 3(a)), while the en face NV-angio area regressed in all patients during and after initial treatment block (Figure 3(b)). After the initial treatment block, en face NV-structure regressed posttreatment by median 15% (range −28%–100%) and mean regression 18.7% ± 39.0% (95% CI 44.2 to −6.8%, *p*=0.26). At the last follow-up, en face NV-structure was regressed by median 6% (range −196%–100%) and mean regression 5% ± 82.7% (95% CI 49.1 to −29.1%, *p*=0.48).

En face NV-angio regressed posttreatment by median 48% (range 11–100%) and mean regression 51.9% ± 29.5% (95% CI 32.6 to 71.2%, *p*=0.007). At the last follow-up, en face NV-angio was regressed by median 34% (range −13%–100%) and mean regression 42.1% ± 39.1% (95% CI 16.6 to 67.7%, *p*=0.015).

#### 3.2.2. En Face Flow Density Measurements

Flow density measurements show a regression within the en face structural area of the NV, while flow density within the en face angio area of the NV showed a less distinct regression (see Table 1 and Figure 2(b)). When measured within the structural NV-area (en face FD-structure, Figure 3(c)), from baseline to posttreatment after the initial treatment block, en face FD-structure decreased from median 72% (range 44–82%) to 38% (range 0–73%), mean decrease of 33% ± 19.2% (95% CI 20.5–45.5%, *p*=0.0077). At the last follow-up, en face FD-structure decreased to median 40% (range 0–70%), mean decrease of 34.3% ± 19.8% (95% CI 21.4–47.3%, *p*=0.0077).

When measured within the NV-angio area (en face FD-angio, Figure 3(d)), from baseline to posttreatment after the initial treatment block, en face FD-angio decreased from median 72% (range 48–89%) to median 58% (range 0–84%), mean decrease of 17.9% ± 25.2% (95% CI 1.4–34.4%, *p*=0.066). At the last follow-up, en face FD-angio decreased to median 54% (range 0–75%), mean decrease from baseline 19.7% ± 22.0% (95% CI 5.31–34.0%, *p*=0.025).

**Figure 4:**
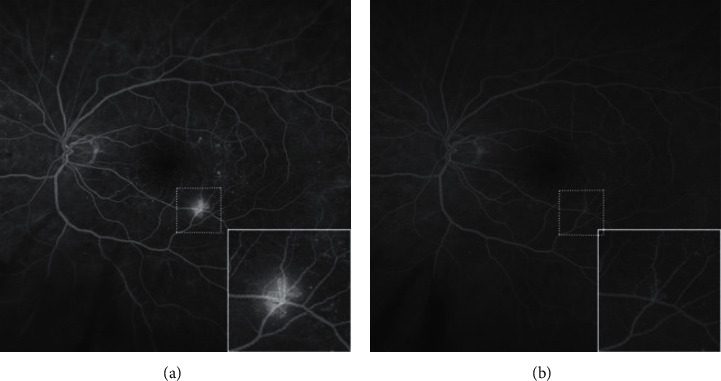
Diabetic neovascularization (NV) of the left eye of a 59-year-old patient with newly diagnosed proliferative diabetic retinopathy on both eyes, with the NV magnified equally (a, b). Initial imaging shows a densely interlaced vascular pattern of the NV with leakage (a) and follow-up imaging after four monthly anti-VEGF injections shows a regressed truncated vascular pattern of the NV without leakage (b), equivalent to column D in Figure 5.

At baseline, most NV showed a densely and interlaced flow structure corresponding to a high vessel density. After treatment, regressed NVs resembled one or several residual truncated main arcs of the previous fan-like vascular structure. Such vascular arcs did not show fluorescein leakage as it is known from active NVs (Figure 4).

#### 3.2.3. B-Scan Structural Images with Flow Overlay

Analysis of B-scan images for structure and flow density showed similar but less distinct changes (Supplementary Figures 1(a)–1(c)).

#### 3.2.4. Visual Acuity and Complications

Visual acuity remained stable within 5 ETDRS letters (or one line on a Snellen chart) with the exception of one patient, whose VA increased 8 and 13 letters (right and left eye, respectively) during the follow-up period. In one eye, a vitreous hemorrhage after treatment discontinuation with anti-VEGF occurred without reduction in VA (see case presentation below). One eye showed diabetic macular edema without sign of recurrent NV, for which anti-VEGF treatment was resumed. No endophthalmitis or other severe complications from intravitreal injection occurred.

### 3.3. Case Presentation

**Figure 5:**
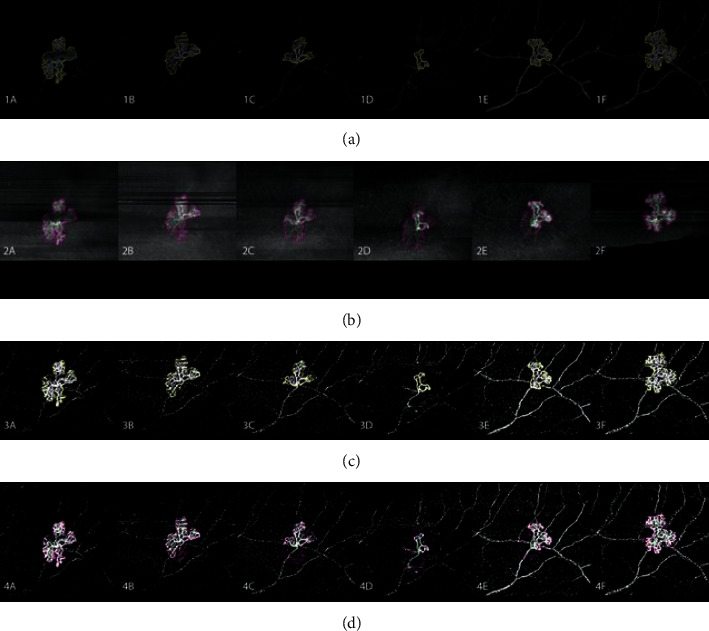
Exemplary follow-up measurements of one neovascularization (NV) with initial regression and later recurrence after cessation of anti-VEGF treatment, as described in the case presentation section, equivalent to the NV in Figure 4 and NV2 in the graphs of Figures 3(a)–3(d). (a) The optical coherence tomography angiography (OCTA) en face angio image with the outline of NV-angio. (b) The OCTA en face structural image with the outline of NV-structure. (c) The binarized OCTA en face angio image with the outline of NV-angio from (a), visualizing flow density (FD) within the angio area (en face FD-angio). While the angiographic size of the NV regresses, the relative flow density remains high. (d) The binarized OCTA en face angio image with the outline of the NV-structure from (b) (en face FD-structure). While the structural area remains relatively stable, its flow density reduces. Recurrence of the NV occurs within the preexisting structural area of the NV. Column A is at baseline, column B is after one week, and column C is one month after the first anti-VEGF injection. Column D represents 22 weeks from baseline, one month after the fourth injection and at the minimal dimensions of NV. Column E shows early recurrence 32 weeks from baseline, 20 weeks after the last injection. Column F shows subtotal recurrence 37 weeks from baseline and 25 weeks after the last anti-VEGF injection, when recurrent vitreous hemorrhage on the fellow eye had occurred, and before retreatment. After retreatment a similar regression was observed.

A 59-year-old treatment-naïve male was diagnosed with bilateral PDR. The small NV was clinically difficult to detect but clearly visible on fluorescein angiography (FA, left eye on Figure 4(a)) and OCTA (Figure 5 column A). He presented with a light vitreous hemorrhage in the right eye caused by a similarly sized NV. In both eyes, one posterior NV could clearly be imaged by 3 × 3 mm OCTA. After informed consent, the patient opted for anti-VEGF treatment with an initial treatment block of four monthly injections in both eyes. The NV regressed in OCTA (Figure 5, row 1) and FA posttreatment showed regression of NV size and leakage in both eyes (Figure 4(b), for the left eye). Upon further observation, slow growth of the NVs could not definitely be detected clinically but was clearly observed in both eyes in OCTA imaging. Hence, retreatment was suggested 20 weeks after the last injection but declined by the patient. A few days later, another light vitreous hemorrhage without reduction of VA occurred in the right eye, and the patient then consented to the resumption of anti-VEGF treatment in both eyes at the next visit, which was 25 weeks after the last injection. A fast regression of the NV was observed, similar to the first treatment response.

In both eyes, size of en face NV-angio area and flow density of en face FD-structure rapidly decreased after the initial treatment block with anti-VEGF. After cessation of treatment, the measurements increased again in both eyes (data series nv1 and nv2 in Figures 2(a)–2(d)). While en face FD-angio showed unchanged flow density in both eyes, en face NV-structure area decreased in one eye and remained unchanged in the eye without vitreous hemorrhage.

## 4. Discussion

As new diagnostic and therapeutic possibilities emerge for PDR, there is the potential for gaining new insights into the pathophysiology of the development of NVs and their response to treatment. This pilot study shows that the structure and flow of NVs respond differently to treatment and can be quantitatively analyzed, followed by repeated OCTA. Also, we propose several interesting imaging aspects for further exploration.

In our study, retinal NVs were observed on OCTA imaging. Retinal NV have been shown to be reliably detectable on OCTA and characterized by shape and assumed origin [13, 17, 27–30]. Russel et al. also found similar progression or regression of NV comparing en face OCTA (12 × 12 mm patterns) with ultrawidefield fluorescein angiography (FA) in a longitudinal series of patients treated with PRP, whereas vascular changes over time were more detailed on OCTA imaging compared to FA [17]. These studies in principle have highlighted the comparability of OCTA with ultrawidefield FA in diagnosis and follow-up of PDR. On the other hand, Schwartz et al. have found B-Scan OCTA to be the most sensitive tool assessing detection rate in NV reactivation [31]. These results highlight the importance of taking into account both planes-en face and B-Scans when using OCTA for follow-up measurements of NV.

Our study has shown that there is a reduction of flow in NVs under anti-VEGF that is sustained over a variable period of time in individual NVs. This confirms existing evidence. Zhang et al. [23] have quantitatively shown regression of neovascularization of the disc in 15 eyes after intravitreal anti-VEGF injection with conbercept. Hu et al. [22] showed a significant decrease in NV vessel length and vessel density within one week of intravitreal conbercept injection compared to untreated patients in a preoperative setting prior to surgical treatment of diabetic neovascular membranes. Elbendary and Abouelkheir [32] have described short-term regression of blood flow in the structural B-scan OCT with overlaid flow information 3 weeks after anti-VEGF treatment of NV of the disc. Ishibazawa et al. have described a rapid regression of flow density after anti-VEGF injection over the optic disc head with recurrence after eight weeks in neovascularization of the disc [14]. He and Yu have described the regression of NV size after PRP and combined anti-VEGF and PRP with similar results to our study [33]. However, the magnitude and sustainability of this flow reduction and what factors may lead to recurrence are still unknown.

To our knowledge, our study is the first to obtain a quantitative combined measure of NV size and vessel density. Also, we assessed the difference of the measurements for the structural and the angiographic areas in the evaluation of NV change under anti-VEGF treatment. Our data showed a much smaller regression of en face NV-structure than that of en face NV-angio. On the other hand, we have shown a much more pronounced regression of flow density within the en face NV's structure compared to the flow density within the detectable NV in the en face angio image. Furthermore, in two cases, we could show detection of reactivation of NV by flow density within the residual en face structural area of the NV, both quantitatively by the increase of FD-structure and qualitatively by the recurrence of perfused vessels within the residual structure.

One could argue that a quantitative measurement of the angiographic NV-outlines is similar and more feasible in clinics. We think taking the structural dimensions of the NV into account allows for more physiological measurements, including the momentarily nonperfused vessels of NV, which can be reactivated in the future. The delineation of the NV in structural en face images is not always easy but very obvious when you look at the B-scan images. Future image analysis methods using the possibilities of artificial intelligence (AI) may allow automated three-dimensional segmentation, detection, analysis, and follow-up of NV in OCTA.

This will open up various prospects for further investigation with regard to the characterization of NVs and early detection of PDR reactivation and retinal NV recurrences, using the full potential of OCTA and clever image analysis. Equivalence of OCTA compared to FA in NV detection has been shown, and future OCTA devices may enable treatment guidance of PDR with OCTA, which may become partially automated. Besides facilitating three-dimensional measurements, there are several advantages to including both structural and flow information in NV analysis. Firstly, as our results show, the change of flow density within the NV's structure seems to be more pronounced than the size change of the NV's angio signal. Secondly, as shown in our presented case, recurrences may be detectable before the occurrence of complications. And thirdly, it is possible to visualize and thus monitor the remaining structure of regressed neovascularizations independent of their current perfusion state, something clinically often referred to as nonperfused vessels, ghost vessels, or fibrotic membranes. VEGF level is a known important factor in the pathogenesis of PDR and effect of its treatment [34]. A changing balance towards vitreous VEGF levels after stopping anti-VEGF injections may lead to reperfusion of previously regressed ghost vessels and thus may be the cause for recurrence of flow in active NV within its preexisting structure. Whether these remnants of vascular structures can permanently occlude remains unanswered by our study. However, the observations of several patients without recurrence of NV during long follow-up periods after stopping anti-VEGF suggest this possibility. This could mean that future therapies might also need to target the residual structural scaffold to prevent recurrences.

Our study confirmed previous observations that regressed NVs remain as a truncated vessel loop not leaking on FA [17]. As discussed by Russel et al., it could be that those larger caliper vessels do not respond as well to blocked VEGF as smaller caliper vessels [17]. However, the implication for recurrence or retreatment of those residual changes remains unclear.

Taken together, the structure of retinal NVs can be measured using OCTA, which, together with its flow (angio) information, might lead to further insight into quantitative analysis and estimation of NV activity after treatment and possibly treatment and retreatment decisions. Based on our findings, we suggest that in the evaluation of retinal NVs, flow density should be measured within the detectable structural area and not only using the en face OCTA angio image.

### 4.1. Limitations

This study has several limitations. Due to its retrospective nature and small sample size, there is some inhomogeneity in initial treatment with three or four anti-VEGF injections and in length of follow-up periods. Measurement pattern sizes were chosen according to clinical needs, which limits the quantitative comparability between patients. A relevant selection bias is introduced to the data. Patients in the working-age population or depending on their ability to keep their driving licence and with presumably good adherence to treatment and follow-up were rather recommended anti-VEGF, while for patients with poor glycaemic control or presumed difficult treatment adherence, PRP was favored, resulting in preferring younger patients with better-estimated treatment adherence and thus possibly better glycaemic control. Also, the need for OCTA as a decision support tool is greater in the case of smaller NV, with possibly less severe PDR. Second, owing to the current technical limitations for OCTA imaging at the time of the study, only the posterior retina until mid-periphery was accessible for OCTA imaging with sufficient image quality for quantitative analysis. Also, all image processing and binarization will introduce imperative biases due to the chosen algorithms. This is inherent to OCTA as an imaging technique based on complex computations of originally measured signals to calculate flow signal and to calculate en face angiographic images, which are then further modified in the process of image analysis, e.g., by binarization [35, 36]. Since angio images are calculated from sequential structural A-Scans, technically, OCTA does not measure actual flow, in contrast to true Doppler-OCT [37]. Another inherent feature of current OCTA devices is their inability to distinguish flow velocity, which is currently addressed in prototype devices or measuring algorithms using variable maybe better variable interscan time analysis [38, 39]. Such principal limitations need to be taken into account in OCTA research. However, current state-of-the-art swept-source OCTA vascular axial and transversal resolution and directional independence surpasses alternative techniques. Moreover, measurement of single B-scan slices shows high variability due to some minor misalignments in follow-up imaging, limiting the observed effects in the B-scan results. Longitudinal graphs may only indicate similar change patterns as described for en face images, but formal quantitative analysis would not be reliable. Furthermore, we have not investigated the role of the posterior vitreous, which has been shown to be of importance as being a scaffold for NV growth, as this lies outside of the scope of this manuscript [40]. Finally, our patients did not routinely receive FA follow-up measurements in the routine clinical setting, so we cannot compare the intensity of the leakage with the NV pattern in the OCTA.

## 5. Conclusions

In summary, our study demonstrates a new quantitative measure of diabetic NV using OCTA. We measure NV size and flow density separately for the structure and angio areas of diabetic NV elsewhere. In our case series, we demonstrate different treatment responses after anti-VEGF between structural and angiographic NV for both area and flow density. The structure remained stable with regression of flow density, while NV-angio regressed with more constant flow density. We thus propose that structural information, which is frequently ignored in the case of OCTA interpretation, should be taken into account in the case of retinal NV, as should be the relation of structure and flow signal. In short, flow density should be measured within the structure of retinal NVs. The rise of AI-based automated image analysis and true three-dimensional structure and flow analysis and faster widefield OCTA will enable better treatment guidance in PDR. Further prospective studies are needed to evaluate clinical benefit for patients, establish reproducible quantitative flow density and retreatment criteria, and compare OCTA to FA as a guiding imaging technique. Our work shows the potential of OCTA in the follow-up of PRD, which is worthy of further investigation.

## Figures and Tables

**Table 1:** Demographics and primary endpoints of changes of diabetic neovascularization measured with optical coherence tomography angiography (OCTA) after intravitreal anti-VEGF.

Patient	Age	Eye	NV#	Previous treatment	Initial treatment	Retreatment/cause	Change (%) of NV-structure size from baseline (=100%)		Chang (%) of NV-angio size from baseline (=100)		Flow density within structural area (FD-structure ) in %			Flow density within angio area (FD-angio) in %			Last follow-up in weeks from baseline (and from last anti-VEGF)		Comments
							NV-structure posttreatment	NV-structure last follow-up	NV-angio posttreatment	NV-angio last follow-up	FD-Structure baseline	FD-structure posttreatment	FD-structure last follow-up	FD-angio postbaseline	FD-angio posttreatment	FD-angio last follow-up	Last clinical follow-up	Last OCTA imaging	
A	59	Right	1	Treatment-naïve	4 monthly IVT	Yes NV recurrance	−51%	−53%	−76%	−83%	64%	38%	28%	81%	69%	59%	46 (0)	46 (0)	Recurrence of NV with vitreous bleeding at week 34 was observed. Rapid regression of NV after retreatment.

A	59	Left	2	Treatment-naïve	4 monthly IVT	Yes NV recurrance	−8%	−3%	−78%	−85%	62%	25%	16%	62%	66%	75%	45 (0)	45 (0)	Retreatment after 37 weeks due to recurrence of NV and vitreous bleeding of fellow eye.

B	30	Right	3	PRP, 3 months before baseline	3 monthly IVT	Yes for DME	−100%	−100%	−100%	−100%	61%	0%	0%	62%	0%	0%	50 (5)	50 (5)	Complete regression after initial treatment block. At weeks 27 and 44 anti-VEGF was administered for DME.

B	30	Left	4	PRP, 10 months before baseline	3 monthly IVT	No	+12%	−27%	−11%	+13%	81%	42%	40%	89%	51%	53%	51 (42)	51 (42)	—
C	48	Right	5	Treatment-naïve	3 monthly IVT	No	+28%	+196%	−65%	−20%	82%	52%	49%	71%	58%	54%	65 (53)	19 (9)	Consistent regression of % flow in structure and % flow in flow area, inconsistent changes of structure and flow area. Stable and clinically inactive PDRP in clinical follow-up for one year after last treatment

C	48	Left	6	Treatment-naïve	3 monthly IVT	No	−15%	−17%	−24%	−38%	79%	26%	41%	82%	36%	57%	64 (53)	23 (13)	Regression of % flow in structure and % flow in flow area, and flow area with grossly unchanged structure area. Partial recurrence in OCTA over time without clinical signs of recurrence for one year after the last treatment.

D	49	Left	7	Treatment-naïve	3 monthly IVT	No	+16%	−6%	−32%	−34%	44%	30%	41%	48%	38%	45%	63 (45)	44 (26)	Regression of % flow in structure and % flow in flow area. Lesser regression of flow. Grossly unchanged structure area, Partial recurrence in OCTA over time without clinical signs of recurrence for 10 months after the last treatment.
																			Previously treated for DME with anti-VEGF.

E	51	Right	8	Treatment-naïve	3 monthly IVT	No	−23%	+52%	−33%	−30%	76%	73%	27%	79%	84%	54%	48 (38)	48 (38)	Delayed reduction of % flow in structure and flow area. Earlier, but inconsistent regression of structure and flow areas. Clinically stable for 9 months after last treatment.

E	51	Right	9	Treatment-naïve	3 monthly IVT	No	−27%	+3%	−48%	−2%	72%	65%	70%	72%	83%	73%	48 (38)	29 (19)	Initial reduction of % flow in structure area and regression of flow area, followed by inconsistent recurrence. Strong fluctuations of structure area and % flow area around the baseline value.

Summary table of all evaluated diabetic retinal neovascularizations (NV), baseline characteristics, and special observations. Measurements at baseline, posttreatment three months after the initial treatment block, and at the final OCTA measurements. Follow-up time for OCTA imaging and clinical follow-up are reported from baseline and from the last anti-VEGF injection. Continuous lines are separating patients, separated lines are separating eyes, and fine lines are separating NV within the same eye. DME = diabetic macular edema; IVT = intravitreal therapy with anti-VEGF (ranibizumab or aflibercept); OCTA = optical coherence tomography angiography; PRP = panretinal laser photocoagulation; RT = retreatment.

## Data Availability

Table 1 shows all primary output measures for the statistical analysis, allowing reproduction of reported statistical analysis and box-plot graphs. The longitudinal graphs represent all measurements. Ground data cannot publicly be released due to local data protection laws, which could possibly allow the deanonymization of single patients.

## Abbreviations

DR: Diabetic retinopathy
PDR: Proliferative diabetic retinopathy
NV: Neovascularization
PRP: Panretinal photocoagulation
VEGF: Vascular endothelial growth factor
DME: Diabetic macular edema
OCT: Optical coherence tomography
OCTA: Optical coherence tomography angiography
FA: Fluorescein angiography.

## Supplementary Materials

Supplementary Materials Supplementary Figure 1(a): longitudinal plot of the height change from baseline (=1) of the protrusion height of the neovascularization (NV) towards the vitreous cavity, measured from the internal limiting membrane to the highest point of flow signal in the B-scan slice with maximal protrusion of the NV. No consistent regression of height can be observed after anti-VEGF treatment. Supplementary Figure 1(b): longitudinal plot of the structural neovascularization (NV) area visible on the OCT in the B-scan slice with maximal protrusion of the NV. Some regression of the structural NV size can be observed in the B-scan, but not across all NVs. Supplementary Figure 1(c): longitudinal plot of the flow density within the B-scan OCT/OCTA with structural information and flow overlay. A reduction of flow density is observed in the B-Scans of all neovascularizations. Nevertheless, the flow density seems to be more variable compared to en face imaging.
