# Supplementary material for: Characterizing Flow and Structure of Diabetic Retinal Neovascularization after Intravitreal Anti-VEGF Using Optical Coherence Tomography Angiography: A Pilot Study
Source: J Ophthalmol. 2021 Jul 14;2021:2942197. doi: 10.1155/2021/2942197 (PMC8294978; doi:10.1155/2021/2942197)
Supplement: Supplementary Materials — Supplementary Figure 1(a): longitudinal plot of the height change from baseline (=1) of the protrusion height of the neovascularization (NV) towards the vitreous cavity, measured from the internal limiting membrane to the highest point of flow signal in the B-scan slice with maximal protrusion of the NV. No consistent regression of height can be observed after anti-VEGF treatment. Supplementary Figure 1(b): longitudinal plot of the structural neovascularization (NV) area visible on the OCT in the B-scan slice with maximal protrusion of the NV. Some regression of the structural NV size can be observed in the B-scan, but not across all NVs. Supplementary Figure 1(c): longitudinal plot of the flow density within the B-scan OCT/OCTA with structural information and flow overlay. A reduction of flow density is observed in the B-Scans of all neovascularizations. Nevertheless, the flow density seems to be more variable compared to en face imaging. [file 2942197.f1.docx]

Supplementary Material


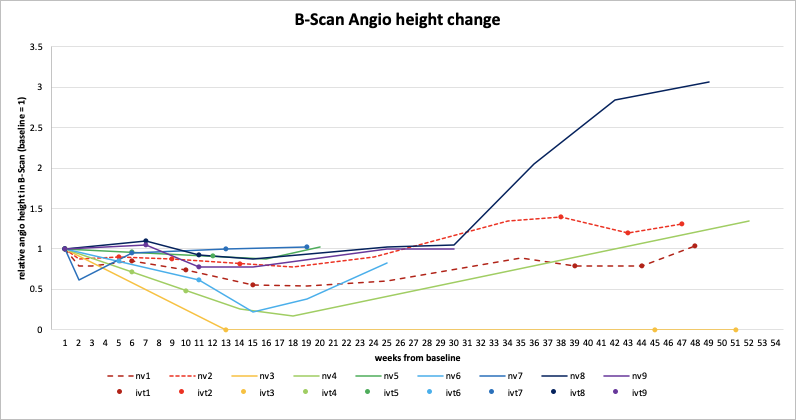


Supplementary Figure 1A: Longitudinal plot of the height change from baseline (=1) of the protrusion height of the neovascularization (NV) towards the vitreous cavity, measured from the internal limiting membrane to the highest point of flow signal in the B-Scan slice with maximal protrusion of the NV. No consistent regression of height can be observed after anti-VEGF treatment.


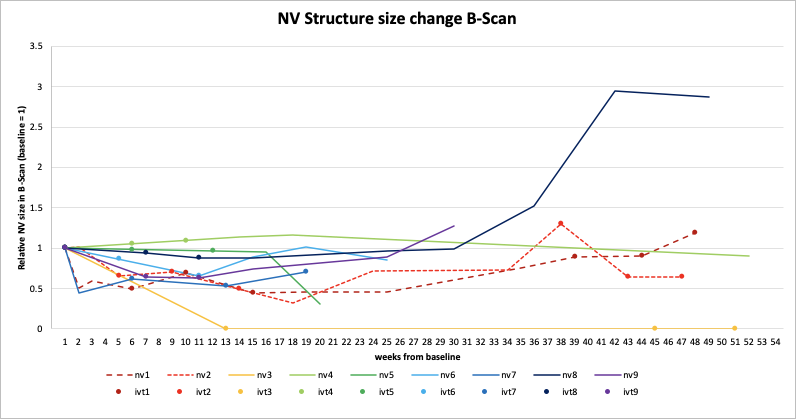


Supplementary Figure 1B: Longitudinal plot of the structural neovascularization (NV) area visible on the OCT in the B-scan slice with maximal protrusion of the NV. Some regression of the structural NV size can be observed in the B-scan, but not across all NVs.


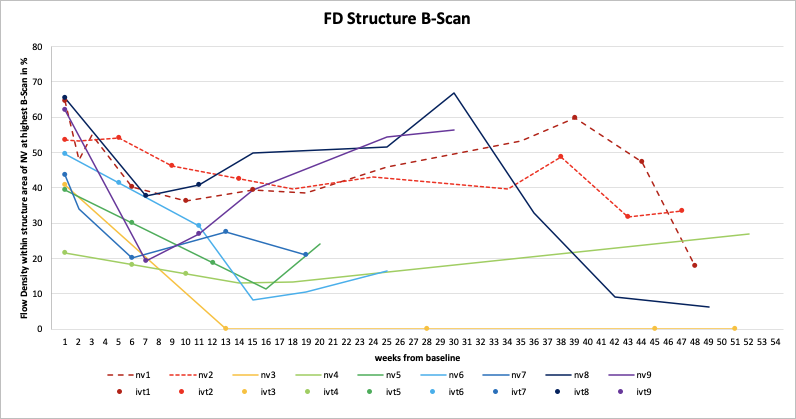


Supplementary Figure 1C: Longitudinal plot of the flow density within the B-Scan OCT/OCTA with structural information and flow overlay. A reduction of flow density is observed in the B-Scans of all neovascularizations. Nevertheless, the flow density seems to be more variable compared to en face imaging.
